# Design and Fabrication of Tubular Scaffolds via Direct Writing in a Melt Electrospinning Mode

**DOI:** 10.1007/s13758-011-0013-7

**Published:** 2012-02-09

**Authors:** Toby D. Brown, Anna Slotosch, Laure Thibaudeau, Anna Taubenberger, Daniela Loessner, Cedryck Vaquette, Paul D. Dalton, Dietmar W. Hutmacher

**Affiliations:** 1 Institute of Health and Biomedical Innovation, 60 Musk Ave, Brisbane, 4059 Australia; 2 Institut für Textiltechnik, Otto-Blumenthal-Str. 1, Aachen, 52074 Germany

## Abstract

Flexible tubular structures fabricated from solution electrospun fibers are finding increasing use in tissue engineering applications. However it is difficult to control the deposition of fibers due to the chaotic nature of the solution electrospinning jet. By using non-conductive polymer melts instead of polymer solutions the path and collection of the fiber becomes predictable. In this work we demonstrate the melt electrospinning of polycaprolactone in a direct writing mode onto a rotating cylinder. This allows the design and fabrication of tubes using 20 μm diameter fibers with controllable micropatterns and mechanical properties. A key design parameter is the fiber winding angle, where it allows control over scaffold pore morphology (e.g. size, shape, number and porosity). Furthermore, the establishment of a finite element model as a predictive design tool is validated against mechanical testing results of melt electrospun tubes to show that a lesser winding angle provides improved mechanical response to uniaxial tension and compression. In addition, we show that melt electrospun tubes support the growth of three different cell types in vitro and are therefore promising scaffolds for tissue engineering applications.

**Electronic supplementary material:**

The online version of this article (doi:10.1007/s13758-011-0013-7) contains supplementary material, which is available to authorized users.

## Introduction

Tubular scaffolds fabricated from electrospun fibers are finding increasing use in tissue engineering (TE) applications, including vascular [1–11] (reviewed in detail by Naito et al.), neural [12–14] (reviewed in detail by Bell et al.) and more recently growth factor delivery [15–19]. Typically, solution electrospun fibers are collected as thin non-woven meshes [20], which has limited their application because the small pore sizes and compromised interconnectivity associated with the random layering of sub-micron diameter fibers acts as a barrier to rather than promotes cell infiltration and subsequent vascularization [21].

For the design and fabrication of tubular scaffolds, control over both the microscopic and macroscopic structure should be reflected in cellular and mechanical responses at varied anatomic locations and biological environments [22]. For example, differences in pressure-dependent mechanical properties between native arteries and artificial grafts induce hydrodynamic flow disturbances and stress concentrations, thereby causing tissue damage as well as impairing cellular function, illustrating the need to match compliance of a designed artificial graft with a small-diameter artery [7]. Whereas rigid nerve guides used in neural TE may cause inflammation due to the stresses exerted by movement, electrospinning allows the fabrication of flexible microfiber and nanofiber guides from the same materials [12]. In bone regeneration, the presence of a solution electrospun tubular scaffold promotes mineralized matrix synthesis, prevents extra-anatomical mineralization, and guides an integrated pattern of bone formation during osteogenic protein delivery in the functional repair of large bone defects in rats [16]. Scaffold morphology which has been studied in terms of tissue growth includes pore size and, more recently pore geometry. Pore size affects cellular activities such as pore spanning (with this ability varying depending on cell type), and the spatial organization of cells within pores. Cell number can also be affected, where a larger pore size often leads to a reduced surface area to volume ratio which then effects cell attachment and proliferation. The effect of pore geometry on tissue growth, such as the influence on tissue orientation and the pattern of tissue growth within pores has been investigated to a lesser extent [23].

What makes this area a particularly promising one is that electrospinning makes it possible to fabricate flexible microfiber and nanofiber tubular scaffolds from polymeric materials, using a broad range of controlled morphologies. In the majority of cases rotating devices are used to fabricate electrospun fibrous tubes. However it is difficult to accurately control fiber arrangements due to the inherent chaotic nature of the electrified jet in solution electrospinning. Although there are approaches for the organized deposition of fibers reported in the literature [22, 24–27], there still remains considerable difficulties in fabricating fibrous tubes with controllable micropatterns [28]. Added to this, charge accumulation effects on solution electrospun fibers tend to restrict the amount of layers which can be collected and remain bound as one coherent structure [29]. As an alternative to solution electrospinning, melt electrospinning permits improved control over the location of fiber deposition and avoids technical difficulties and cytotoxicity concerns associated with the removal and the presence of residual of solvent [24, 30].

In contrast, our group has shown most recently that scaffolds with a thickness of up to 1 cm can be designed and manufactured with controlled architectures in a direct writing mode, demonstrating that melt electrospun fibers can be readily placed on top of each other, as the deposited fibers have minimal residual charge [31]. In this work, we demonstrate melt electrospinning in a direct writing mode onto a rotating cylindrical collector. This allows the design and fabrication of tubular scaffolds of any size using micron diameter fibers, with controllable microscopic and macroscopic architectures and mechanical properties. A key design parameter is the fiber winding angle, which allows control over scaffold pore morphology (e.g. size, shape, number and porosity) and influences the mechanical properties of such a tube. Furthermore, we establish a finite element (FE) model which can be used as a predictive design tool, where in this case the simulated response to uniaxial loading of simplified tubular structures varies when the fiber angle is altered. This response is validated against mechanical testing results of fabricated electrospun tubes. We further show that the electrospun tubes support the growth of three different cell types in vitro and are therefore promising scaffolds for TE applications.

## Experimental

### Experimental Materials

Poly(ε-caprolactone) (PCL) with M_W_ = 50 kDa was kindly supplied by Perstorp, UK Ltd. (United Kingdom) and used as received. The superior rheological and viscoelastic properties within the group of aliphatic polymers makes PCL the polymer of choice to be applied during the development of scaffolds based on melt extrusion processes. Coupled with relatively inexpensive production routes and FDA approval, this provides a promising platform for the production of longer-term degradable and biocompatible implants which may be manipulated physically, chemically and biologically to possess tailorable degradation kinetics to suit a specific anatomic site as reviewed in detail by Woodruff and Hutmacher [32].

### Fabrication of Tubes

#### Melt Electrospinning

PCL pellets were loaded into a plastic Luer-lock 3 mL syringe (B-Braun, Australia). The syringe was placed in a custom glass water jacket housing (Labglass, Australia) through which heated water at 78°C was circulated using a recirculating water tank (Ratek, Australia). The syringe was heated for 1 h to provide a homogenous polymer melt. A 21 G hypodermic needle (Becton–Dickinson, Australia) with the tip flattened was attached to the syringe to be used as the spinneret. The feeding rate of the polymer melt in the syringe was controlled at 50 μL/h using a programmable syringe pump (World Precision Instruments, USA). A high voltage (Emco High Voltage Corporation, USA) of 12 kV was applied to the needle and the distance between the tip of the needle and the rotating collector was 40 mm.

#### Single Fiber Production

To produce single melt electrospun fibers, the electrospinning settings described above resulted in an average fiber diameter of 60 μm. The fibers were collected on a flat collector translating at 250 mm/min to draw them into a straight line.

#### Tube Winding

A 6 mm outer diameter brass tube connected to a stepper motor to provide rotation was used as a grounded collector, mounted on top of a linear slide (Velmex Inc., USA) to provide lateral translation in one direction. Custom translation patterns were written in G-code and controlled using Mach3 motion control software (Artsoft, USA). Different combinations of lateral translation and rotation speeds of the tube resulted in a varied effective tangential vector, comprised of a winding speed and winding angle (i.e. the angle between the fiber direction and the axis of mandrel rotation). Table 1 shows that different combinations of rotational and translational speed could be used in order to achieve a desired effective winding angle. Two cases are shown to achieve angles of 30°, 45° and 60°. In case 1 the translation speed is held constant, while in case 2 the rotational speed is fixed to maintain a constant tangential speed. In each case, the stage was programmed to translate back and forth 700 times to create 700 fiber pairs, so that there was a sufficient structure for subsequent mechanical testing. Five samples were fabricated for each case shown in Table 1. By automating the process, batches of five tubes were wound on the same collector using constant electrospinning parameters. Prior to mechanical testing, each sample was weighed using an AUW220D UniBloc Balance (Shimadzu, Australia).

**Table 1:** Parameters used to fabricate melt electrospun tubes with different winding angles

Case	Translational speed (mm/min)	Effective translational speed (mm/min)	Number of collector rotations/translation	Tangential speed (mm/min)	Resultant vector speed (mm/min)	Effective resultant vector speed (mm/min)	Actual winding angle (°)	Effective winding angle (°)	Measured winding angle (°)
1a	3,500	637	0.306	368	3,519	735	6	30	29.3 ± 2.3
1b	3,500	637	0.531	637	3,557	901	10.3	45	45.4 ± 2.2
1c	3,500	637	0.919	1,103	3,670	1,274	17.5	60	58.3 ± 3.0
2a	6,062	1,102	0.306	637	6,095	1,273	6	30	29.1 ± 2.8
2b	3,500	637	0.531	637	3,557	901	10.3	45	41.1 ± 4.8
2c	2,021	368	0.919	637	2,119	735	17.5	60	59.0 ± 2.8

### Mechanical Testing

Mechanical testing of single fibers in tension, as well as tubes in tension and compression was undertaken. An Instron 5848 Micro Tester with a 5 N load cell (Instron, Norwood, MA) was used to apply static forces. The rigid load frame was configured vertically to suit each testing case, with the set up consisting of mounted grips or plates to fix the specimens between the base beam and the movable actuator [33]. In each tube testing case, displacement was applied and the reaction force measured by the 5 N load cell recorded using Instron WaveMaker Runtime 32 software (Instron, Norwood, MA). Single melt electrospun fibers were placed between air pressure grips separated by 10 mm and extended up to 225% at a 1%/s rate at room temperature (*n* = 10) [34]. Compression and tension tube tests were performed for cases 1a–1c, by applying a 10% deformation with a strain rate of 1%/s (*n* = 5) [34]. For tensile testing of tubes, small plastic 6 mm diameter cylinders (2 mm thick) were glued to the top of modified syringe plungers for fixation to air pressure grips in the Instron Micro Tester. Each end of a tubular sample was bonded using epoxy glue to a fixation cylinder and then mounted in the Micro Tester using compressed air clamps. For compression, tubular samples were placed between a flat plate and a 10 mm diameter cylinder mounted on the moving actuator, so that the axis of the tube was in line with the central axis of the moving actuator.

### Characterization

For morphology observation, scaffold surfaces were sputter coated with gold (a 10 nm thick layer) using a Leica Microsystems EM SCD005 (Germany) and scanning electron microscopic (SEM) examination carried out using an FEI Quanta 200 Environmental SEM (Netherlands) at an accelerating voltage of 10 kV. Images were prepared and processed using Corel DRAW X4 and PHOTO-PAINT X4 (Corel Corporation, Australia) and Adobe Photoshop and Illustrator CS4 (Adobe Systems Incorporated, Australia) using image auto-adjust functions. For fiber diameter measurements, five images were taken at randomly chosen locations for each winding angle case. Images were imported into ImageJ 1.41 software (NIH, USA) and ten fiber diameter measurements taken for each fiber orientation. Similarly on these images, for pore size measurements a polygon was drawn along the edges of four intersecting fibers to define the outer bounds of a void (or pore) created by the fibers [23]. For porosity measurements, a middle 3 mm section of each tubular sample was analyzed using micro computed tomography (μCT). Specimens were scanned on a Scanco μCT40 (Scanco Medical AG, Switzerland) at 6 μm resolution, employing 55 kV and 145 μA with 500 ms integration time. After segmentation, a bone morphometric analysis algorithm was used to calculate porosity as the ratio of the material volume to the total volume scanned. The mean of measured values, standard error of the means, and differences between the means (using one-way ANOVA with the assumption of normally distributed data) were then calculated using PASW Statistics 18 software (SPSS Inc., USA). Optical images of tubes and experimental conditions were taken using a Canon EOS 450D digital single lens reflex (DSLR) camera with a Canon EF-S 17-85 mm USM lens (Canon.Inc, Australia).

### Finite Element Modeling

#### Preparation of Model for Mechanical Testing Simulation

Tube geometry was prepared in ANSYS^®^ Mechanical APDL, Release 12.1 (ANSYS Inc., USA). To simulate the winding process, helical lines were created in pairs representing one back and forth translation of the rotating mandrel on which electrospun fibers were collected. Each fiber pair originated at the same location, with one helix line directed clockwise and the other counter clockwise. For computational efficiency, ten fiber pairs were distributed equally around the circumference of a 6 mm diameter circular base (assuming equally distributed fibers). Multiple geometries were created where the winding angle was varied from 15° to 85° [35], however in each case the total tube height was 10 mm.

#### Preprocessing

Three noded quadratic BEAM188 elements with a circular cross section of 60 μm in diameter (resultant diameter of single fiber fabrication) were used to mesh the geometry. Enabling shear stress calculations through the elements by using Thimoshenko beam theory, the BEAM188 element is the most accurate beam element provided by ANSYS 12.1 and suits the fiber geometry in this model [36]. In order to accurately represent the circular structure, the elements were used in their quadratic form. Following refinement analysis, the division arc spanned by one element was defined to be not bigger than 3° to be able to approximate the circular structure using straight elements [37]. The helical lines were defined for two cases: independent of each other (not connected) to obtain information about influence of winding angle on the stability of the geometry in terms of single fibers; and alternatively, contact between the elements where the helical lines crossed each other was defined as bonded to represent fusion between the fibers during melt electrospinning.

#### Material Model

**Fig. 1:**
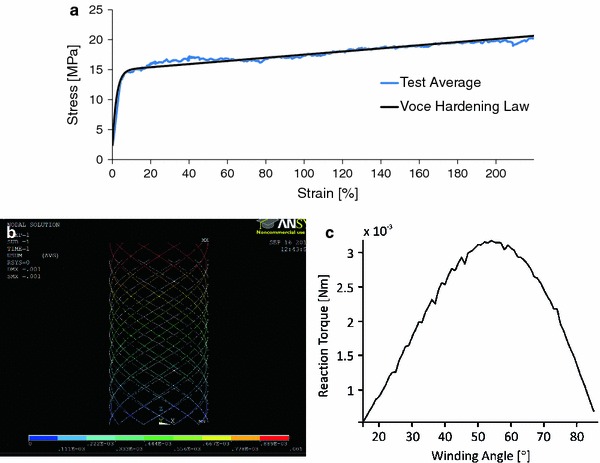
**a** Comparison of mechanical testing single fiber properties for PCL with Voce hardening law. **b** Example of tube model subjected to compressive loading in ANSYS. **c** Simulated response to torsional loading as winding angle is varied

To develop a material model representing melt electrospun PCL fibers, the linear region of the stress–strain curve of the averaged data from the single fiber tensile testing was used. The Young’s Modulus was approximated to be 0.35 GPa, with a yield stress of approximately 13 MPa at 4% strain. This result matches values previously reported in the literature [38]. Additional material properties used to represent PCL included a Poisson’s ratio of 0.47 [39] and a density of 1.145 (g/cm³) [40]. ANSYS provides different nonlinear material models, but only the Voce Hardening law describes a continuous stress–strain curve approximation depending on four material specific parameters, leading to more accurate results than a multilinear approximation [41]. With the Marquardt–Levenberg-Algorithm the four material parameters defining the Voce hardening law were determined to be*k* = 2.39 × 10^6^,*R*_0_ = 2.62 × 10^6^,*R*_inf_ = 1.24 × 10^7^,*b* = 160. A test simulation repeating the single fiber tension test represented by a single Beam188 element of 10 mm length, fixed at one end and extended in steps of 0.1 up to 25 mm was carried out to test the applied material law. A comparison of the mechanical testing and simulation results can be seen in Fig. 1a.

#### Loading

In each simulation case, all nodes at*z* = 0 (bottom of the tube) were constrained in all degrees of freedom (fixed supports). All nodes located at*z* = 10 mm were loaded with a deformation of ±1 mm parallel to the axis of symmetry to simulate 10% uniaxial tension and compression (Fig. 1b). For model validation, each geometry variant was rotated 45° about the central axis of the tube (torsion) (Fig. 1c).

#### Postprocessing

The reaction forces at the end of each fiber (*z* = 10 mm) were added, giving a total reaction force in the tensile and compressive loading cases. The total torsion reaction was calculated by transforming the resultant x and y directed forces into radial and tangential forces, then summing and dividing them by the associated arc length.

### In Vitro Biocompatibility Assessment

Primary human osteoblasts (hOBs) and mesothelial cells as well as mouse osteoblasts (mOBs) were utilized in this study in order to demonstrate that the electrospun fibers comprising the tubes are non-cytotoxic and support cell colonization.

#### Biomimetic Coating of PCL Tubular Scaffolds for In Vitro Studies with Osteoblasts

To enhance osteo-induction, PCL tubes were coated with a layer of calcium phosphate (CaP). The coating process consisted of three steps: surface activation with alkaline treatment [Sodium hydroxide (NaOH)]; treatment with simulated Body Fluid 10× (SBF10×) to deposit the CaP; and post-treatment with NaOH. The preparation of SBF10× was adapted from Yang et al. [42]. For 1 L of solution, reagents were dissolved in ddH20 in the following order: 58.430 g NaCl, 0.373 g KCl, 3.675 g CaCl_2_·2H_2_O and 1.016 g MgCl_2_·6H_2_O. The next reagent (1.420 g of Na_2_HPO_4_) was dissolved separately in 20 mL of ddH20 and added drop by drop into the main solution while maintaining the pH level at 4 by adding hydrochloric acid (HCl) 32% in order to avoid precipitation of calcium cations and phosphate anions. The tubes were first cleaned by immersion in 70% ethanol solution under vacuum for 15 min for the purpose of removing entrapped air bubbles, then the structures were immersed into pre-heated (37°C) NaOH 2 M and a 5 min vacuum treatment was performed at room temperature. For the rest of the activation step, the scaffolds were placed at 37°C for 30 min to accelerate the etching process. The tubes were then rinsed with ddH2O until the pH level dropped to approximately 7. Meanwhile, NaHCO_3_ was added to the SBF10× solution until a pH of 6 was reached. This activated SBF solution was filtered (0.2 μm filter) and another 5 min vacuum treatment at room temperature was performed to ensure that the solution fully penetrated the tubes. The samples were thereafter placed at 37°C for another 30 min. The solution was replaced with freshly activated and filtered SBF and placed again at 37°C for 30 min. The tubes were rinsed in ddH2O and then immersed in pre-heated NaOH 0.5 M for 30 min at 37°C. Finally the tubes were rinsed with ddH2O until the pH level dropped to approximately 7 and then dried overnight in a dessicator.

#### Human Primary Osteoblast Cell Culture and Seeding onto PCL Tubular Scaffolds

hOBs were isolated as previously described [43]. Cells were maintained in culture up to passage 4 in alpha minimum essential media (α-MEM) (Invitrogen, Australia) supplemented with 10% FBS (Invitrogen, Australia), 100 IU/mL penicillin (Invitrogen, Australia), and 0.1 mg/mL streptomycin (Invitrogen, Australia). Prior to cell seeding, PCL tubes were sterilized by immersion in 70% ethanol for 30 min, followed by 20 min UV exposure. Dry scaffolds were transferred into a 24-well plate and 1.6 × 10^5^ hOBs suspended in 40 μL of culture media were equally distributed onto the scaffolds. After leaving the moistened scaffold for 2 h at 37°C, during which initial cell attachment to the scaffold occurred, 1 mL of culture medium was carefully added. Cells were grown over a total culture period of 4 weeks in 5% CO_2_ and at 37°C. Medium was changed every 3 days. After 2 weeks of culture, cells were cultured under osteogenic conditions (i.e. in cell culture medium supplemented with 50 μg/mL ascorbate-2-phosphate (Sigma-Aldrich, Australia), 10 mM β-glycerophosphate (Sigma-Aldrich, Australia), and 0.1 μM dexamethasone (Sigma-Aldrich, Australia)).

#### Mouse Primary Osteoblast Cell Culture and Seeding onto PCL Tubular Scaffolds

MOBs were received as a gift from Dr. Jean-Pierre Levesque (Mater Medical Research Institute, Brisbane, Australia). Cells were maintained in culture in α-MEM (Invitrogen, Australia) supplemented with 10% FBS (Invitrogen, Australia), 100 IU/mL penicillin (Invitrogen, Australia), 0.1 mg/mL streptomycin (Invitrogen, Australia) and 50 μg/mL ascorbate-2-phosphate (Sigma-Aldrich, Australia). Prior to cell seeding, PCL tubes were sterilized by immersion in 70% ethanol for 30 min, followed by 20 min UV exposure. Dry scaffolds were transferred into a 24-well plate and 3 × 10^5^ mOBs suspended in 37.5 μL culture media were equally distributed onto the scaffolds. After leaving the moistened scaffold for 2 h at 37°C, during which initial cell attachment to the scaffold occurred, 1 mL of culture medium was carefully added. Cells were grown over a total culture period of 4 weeks in 5% CO_2_ and at 37°C. Medium was changed every 3 days. After 2 weeks of culture, cells were cultured under osteogenic conditions, i.e. in cell culture medium supplemented with 50 μg/mL ascorbate-2-phosphate (Sigma-Aldrich), 10 mM β-glycerophosphate (Sigma-Aldrich), and 0.1 μM dexamethasone (Sigma-Aldrich).

#### Culture and Seeding of Human Mesothelial Cells onto PCL Meshes

Human mesothelial Met-5A cells were obtained from the American Type Culture Collection (ATCC) and maintained in the recommended media199 [44]. Briefly, 2 × 10^5^ cells were seeded on top of sterilized PCL meshes, grown for up to 28 days and processed using confocal laser scanning microscopy (CLSM) as described previously [45].

#### Analysis of Cell Viability

After 28 days of 3D culture, cell viability was assessed by fluorescein diacetate (FDA) (Invitrogen, Australia) and propidium iodide (PI) (Invitrogen, Australia) staining. Samples were rinsed three times with pre-warmed phenol red free α-MEM (Invitrogen, Australia). Samples were then incubated in 2 μg/mL FDA and 10 μg/mL PI in phenol red-free medium at 37°C for 15 min and washed using phosphate buffered saline (PBS). Thereafter, the hydrated specimens were immediately imaged using a confocal microscope (TCS SP5 II, Leica, Australia) with a 10/20× immersion oil objective. 2 μm thick Z–stacks were acquired over a total height of 400–600 μm and from these, 3D projections were generated using LAS AF software (v.1.8.2 build 1465, Leica Microsystems, Australia). Then, the scientific image processing software Imaris (x64; v.7.3.0, Bitplane, Switzerland) was used to prepare 3D reconstructions.

#### Phalloidin/DAPI Staining of Cells Growing on PCL Scaffolds

Cells were fixed with 4% paraformaldehyde for 20 min at room temperature (RT) and permeabilized using 0.2% Triton X-100 in PBS for 5 min at RT. Thereafter, samples were washed twice with PBS and blocked for 10 min in 2% bovine serum albumin (BSA) (Sigma, Australia) in PBS. Samples were then incubated with 5 μg/mL 4′,6-diamidino-2-phenylindole (DAPI) (Invitrogen, Australia) and 200 U/mL rhodamine-conjugated phalloidin in 2% BSA/PBS for 1 h at RT. After washing with PBS, hydrated samples were imaged using a laser scanning confocal microscope (TCS SP5 II, Leica). Z-stacks and 3D projections were prepared as described above.

#### SEM Analysis of Cells Growing on PCL Scaffolds

Samples were fixed with 3% (v/v) glutaraldehyde in 0.1 M sodium cacodylate buffer (pH 7.3) overnight at 4°C. Fixed specimens were washed in 0.1 M sodium cacodylate buffer and dehydrated through a graded series of ethanol. Samples were then incubated in hexamethyldisilazane (HMDS) (Prositech, Australia) for 30 min and air-dried. Specimens were mounted and gold coated (SC500, Bio-Rad, Australia) prior to visualization using a Quanta 200 SEM (FEI, The Netherlands).

## Results and Discussion

### Design of Porous Tubular Structures

**Fig. 2:**
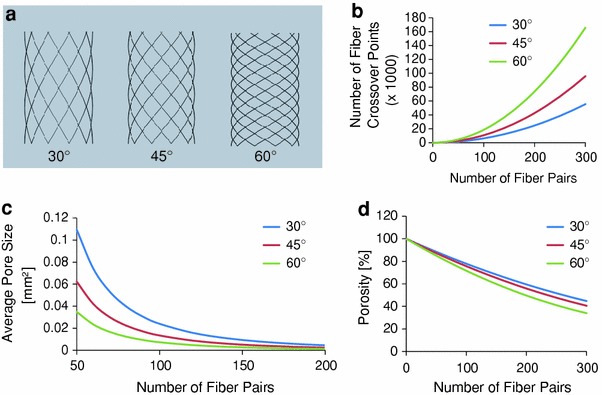
**a** Variation in pore architecture and quantity with winding angle for ten fiber pairs. Increase in number of fiber crossover points (**b**) and corresponding decrease in average pore size (**c**) with increasing number of fibers and winding angle. **d** Variation of total porosity with number of fiber pairs and winding angle

Modifying design parameters such as the fiber diameter, number of fibers and the choice of winding angle allows control over the spatial architecture of a tubular scaffold fabricated from direct writing combined with melt electrospinning. For example, Fig. 2a illustrates the variation in pore size and number, geometry and orientation, as well as fiber crossover points when the winding angle is varied from 30° to 45° and 60° respectively for a 6 mm diameter, 10 mm high tube designed from ten fiber pairs on a single layer. In this case the pore geometry varies (left to right) from a diamond shape with the smaller angles oriented axially, to a square, to a diamond oriented radially. In addition, the number of equivalent full pores/fiber crossover points increases with a larger winding angle from (left to right) 60/70, 90/100, to 180/190 respectively (where in any case the number of fiber crossover points will be greater than the number of pores by the amount of fiber pairs chosen).

For a single layer of wound fibers with diameter of 25 μm, Fig. 2b demonstrates a power law relationship between the number of fiber pairs (as well as the number of pores) and the number of crossover points, which increases with a larger winding angle. Associated with this increase in number of crossover points/pores with more fiber pairs is a decrease in average pore size (assuming a homogenous distribution of fibers) which becomes more apparent with increasing winding angle (Fig. 2c).

Assuming a fixed fabrication time, a constant amount of material should be collected. However as the winding angle increases, so will the length of a single fiber from one end of the tube to the other (assuming the tube height is fixed). Therefore, this will be accompanied by an associated decrease in fiber diameter to maintain a constant volume of material. As well as showing the reduction in porosity as the number of fibers increases, Fig. 2d illustrates a decrease in porosity with increasing winding angle. This can be explained by the reduced volume occupied by the smaller diameter fiber associated with increased winding angle.

The relationships between the parameters described above are presented in the supplementary information. This allows the design of tubes with control over features such as pore size, shape and number as well as total porosity, based on the following input parameters: tube height and diameter, fiber diameter, number of fiber pairs, and winding angle. Their choice and balance between them allows the tailoring of the pore architecture to suit a specific TE application. For example, for a desired pore size and using a fixed number of fibers, a large winding angle such as 60° allows for an increased number of crossover points (for tissue spanning) and surface area to volume ratio than compared to a more acute angle. The associated decrease in pore size reduces the potential for cell and tissue infiltration which may be favorable for example to neural compared to vascular applications.

### Mechanical Simulation

**Fig. 3:**
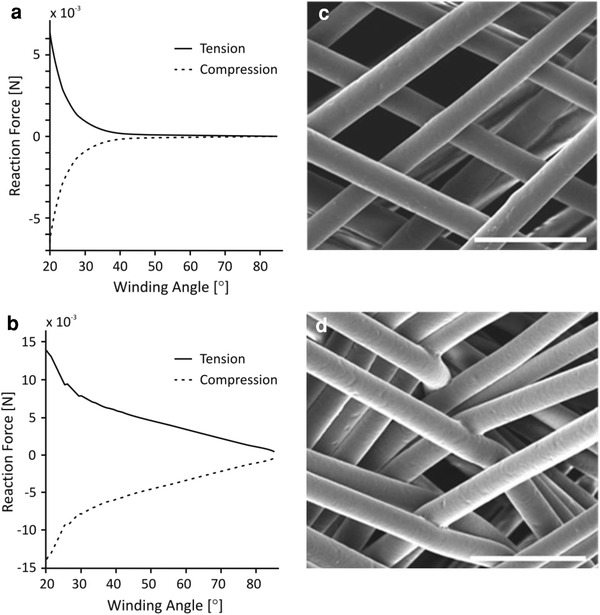
Comparison of simulated response to uniaxial tensile and compressive loading for tubes comprised of ten **a** unbonded and **b** bonded fiber pairs with varying winding angle. SEM images showing interwoven melt electrospun fibers with **c** minimal evidence of bonding due to melt fusion at the crossover points, and **d** evidence of fusion between fibers. All *scale bars* 100 μm

The development of a static structural computational model allows the analysis of different loading scenarios leading to improved design of TE scaffolds. For example, for the simple case of axial loading we hypothesized that the stiffness of a tubular construct would be influenced by varying a design parameter such as winding angle (Fig. 3a, b) due to two mechanisms: the degree of initial relative fiber alignment to the direction of loading; versus the number of fiber crossover points which provide sites for bonding. Insufficient computational power was available to simulate a complex tubular structure composed of 700 fiber pairs (as was fabricated), due to the number of elements required to represent the geometry. One method investigated to simplify the tubular geometry was to reduce the number of fiber pairs to ten. At this stage of development, in order to quantitatively predict the stiffness response of a 700 fiber pair tube by normalizing against 10 fiber pairs would require further simulations with increased numbers of fiber pairs to establish the nature of the variation in response (i.e. linear or non-linear). However, the established model still allows a qualitative identification of response trends which can be compared with experimental results. For preliminary validation, the winding angle which yielded the optimal response of the model to torsional loading (Fig. 1c) matched the reported angle of 54°–55° traditionally used in filament winding processes [46].

It has been observed that melt electrospun fibers may solidify prior to deposition (Fig. 3c) or alternatively, fuse at the points of contact if insufficient cooling has taken place prior to deposition (Fig. 3d). Matsuda et al. reported an increase in stiffness of an electrospun tubular construct with an increased degree of fusion or welding at the contact points with other fibers, attributed to the restricted freedom of movement of fibers under mechanical stretching. Therefore, a physically entangled mesh should exhibit a higher degree of fiber extension upon stretching than with bonded fibers [7].

For a melt electrospun tube produced in a direct writing mode with the same number of fiber pairs, a larger winding angle will facilitate more fiber bonding sites. However, for the loading cases of uniaxial tension and compression, this could be accompanied by a sacrifice in the stiffness of the construct as the orientation of the fibers diverges further from the direction of loading. Figure 3 illustrates the results of simulated uniaxial tensile and compressive loading of a tubular construct for two cases: where there is no bonding between fibers (Fig. 3a), and where the fibers are fully bonded (Fig. 3b). These results show an improvement in stiffness when the fibers are bonded compared to unbonded. In the unbonded case (Fig. 3a), stiffness reduces as the winding angle is increased from 30° to 60° according to a power law. Whereas in the bonded case (Fig. 3b), stiffness reduces linearly as the winding angle is increased from 30° to 60°. However, the fact that stiffness continues to reduce with increasing winding angle for the bonded case suggests that the orientation of fibers closer to the axis of loading improves stiffness more significantly than the increased amount of fiber bonding sites provided by fibers orientated further from the axis. Furthermore, the nature of the difference between the stiffness response (power law vs. linear) with varied winding angle to such loading cases may be used to provide an indication as to the degree of fusion between fibers in the constructs.

A limitation of this model is shown by the fact that the compressive and tensile results are equal, due to the linear approximation for the material model based on tensile testing results. With further development, such as the inclusion of compressive material properties (which would account for failure of slender fiber segments in compression due to buckling as observed in Fig. 6b) as well as cyclic loading cases (to better represent in vivo loading) such a predictive design tool could be applied to more complicated combined variable loading cases, which may benefit from the design of structures with increased complexity, such as multi-angle wound tubes.

### Fabrication of Melt Electrospun Tubes in a Direct Writing Mode

Melt electrospinning offers the advantage of a stable jet which allows relatively good control over the location of fiber deposition [47]. However, on a stationary collector fiber deposition remains random. The melt electrospinning parameters previously described resulted in fibers collected with an average diameter of 60 ± 1.1 μm on a stationary collector. We have previously shown that melt electrospun fibers can be drawn into a straight line when relative motion is introduced into the collector at speeds matching or above the jet speed [31]. For a fiber diameter of 60 μm, a relative collector speed of approximately 250 mm/min was required to draw the fiber into a straight line. Increased speeds are observed to introduce a drawing effect onto the fibers which reduces fiber diameter and has been reported to improve mechanical properties [48]. To obtain the benefits of ultrafine fiber diameters associated with electrospinning, the relative collector speeds were chosen at above the jet speed, in order to further reduce the collected fiber diameter. Table 2 shows that fiber diameter reduces with increased collector speed. Therefore, in this work melt electrospinning offers a 25 times reduction in fiber diameter from 514 μm (the inner diameter of the needle through which the polymer melt was extruded) down to 20 μm, due to the combination of electrostatic drawing and further mechanical drawing associated with the winding process. This is an order of magnitude reduction in fiber diameter compared to melt spinning processes where the electrostatic drawing element is absent [49].

**Table 2:** Fabrication times and resultant dimensions for two different strategies to fabricate melt electrospun tubes

Case	Fabrication time (min)	Effective resultant vector speed (mm/min)	Weight (mg)	Average fiber diameter (μm)
1a	22	735	15.2 ± 3.3	25.7 ± 1.8
1b	22	901	14.8 ± 2.5	22.4 ± 1.8
1c	22	1,274	15.7 ± 2.9	19.9 ± 0.9
2a	13	1,273	11.1 ± 1.2	20.4 ± 1.8
2b	22	901	17.5 ± 0.7	26.3 ± 3.2
2c	38	735	37.4 ± 1.3	27.7 ± 2.4

#### Collection Speeds

**Fig. 4:**
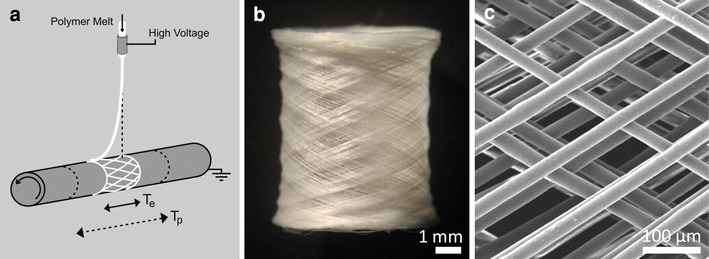
**a** Schematic illustration showing fabrication of a porous tube by combining melt electrospinning with direct writing by using a rotating collector on a translating stage. Due to the characteristic lag effect of the melt electrospinning jet, the effective translation of the jet (T_e_) which corresponds to the tube height is shorter than the actual programmed translation of the stage (T_p_). **b** Porous tube fabricated by combining melt electrospinning of PCL with direct writing. **c** SEM image showing smooth uniform fibers interwoven and oriented at 60° to the central axis of the tube

Two strategies were employed to obtain the effective resultant vector speeds shown in Table 1. Both cases combined lateral translation with rotation of a cylindrical collector (Fig. 4a). In the first case the programmed lateral translational speed (T_p_) was held constant at 3,500 mm/min. One phenomenon we have previously described is an associated tensile drag force imparted on the melt electrospinning jet as it collects onto surfaces moving at relative speeds greater than the jet speed, causing the collected fiber to experience a delay in response (“lag”) to changes in direction (Fig. 4a). This lag effect increases as the collector speed is further increased [31]. In this case, when the collector was programmed to translate back forth over a distance of 55 mm, due to a delayed turning response the resultant collection range was over 10 mm (the desired height of the tube). Therefore, the effective translation speed (T_e_) was calculated as the ratio of actual collection length to programmed length multiplied by T_p_. Rotation was then combined with translation. A ratio of rotational to translational speed was programmed to obtain a tangential speed, which through vector addition resulted in a vector speed and actual winding angle. However, taking into account the lag effect on the melt electrospun fiber, the effective resultant vector speed and angle were obtained by combining the effective translational and tangential speeds. Thus, for a constant translational speed, by varying the tangential speed the resultant winding angle could be controlled. The difference between the measured winding angles and actual winding angles due to the lag effect is shown in Table 1. Figure 4b and c shows a 6 mm diameter tube with a controlled winding angle of approximately 60°.

By maintaining a constant T_p_ in case 1, the fabrication time (to translate back and forth 700 times) was fixed. This should ensure a constant amount of material collected. Table 2 shows that for a fabrication time of 22 min the weight of tubes fabricated in this manner was relatively uniform. However, because the length of a single fiber from one end of the tube to the other will increase with an increase in winding angle, this should be accompanied by an associated decrease in fiber diameter to maintain a constant volume, shown in Table 2.

As an alternative method to control the winding angle, the tangential speed was fixed and T_p_ varied. The tangential speed of 637 mm/min used in case 1 for a 45° winding angle was chosen as a reference point so that the translational speed could be varied above and below the translational speed matching this speed to obtain winding angles of 30  and 60° (Table 1). However, an approximate 1.7 fold increase and decrease in the translational speeds was required to achieve the desired changes in winding angle. Because of the associated differences in fabrication time and total volume of the tubes fabricated in this manner (Table 2), this method was not preferred for subsequent characterization and comparisons of mechanical properties based on winding angle.

#### Pore Size and Porosity

**Fig. 5:**
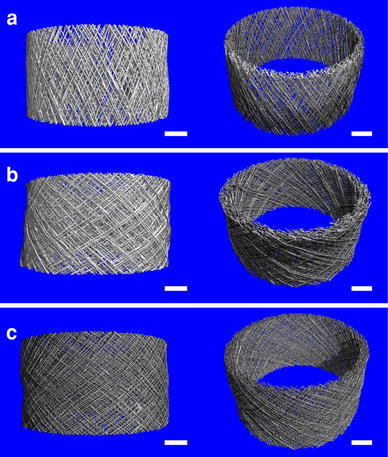
Front and oblique μCT images of a 3 mm central section of melt electrospun tubes shows tubes are comprised of ten fibrous layers and the pore size decreases as the winding angle is increased from **a** 30° to **b** 45° and **c** 60°. All *scale bars* 1 mm

Figure 5a–c shows μCT images of the middle 3 mm sections of tubes with 30°, 45° and 60° winding angles respectively. Upon inspection, each tube appears comprised of 10 layers of fibers, that is, 70 fiber pairs per layer. Assuming a fiber diameter of 25.7 μm for the 30° case (based on the average measured value in Table 2), to maintain a constant volume fiber diameters of 23.23 μm and 19.53 μm would be required for the respective 45° and 60° cases (where these values fall in the range of measured values in Table 2). Using these values, predicted values for porosity were established using the formula presented in the supporting information for two cases: assuming no bonding between fibers, and assuming total bonding between fibers at the crossover points on a single layer (Table 3). Assuming no bonding between fibers the porosity is predicted to be greater than for the bonded case, where in both cases the predicted porosity decreases with increasing winding angle. For measured porosity using μCT analysis, there was little variation as winding angle was varied, though the values lay in between those predicted for the unbonded and bonded cases. However, the measured values lie closer towards those for the unbonded case suggesting there is a minor degree of fusion between fibers, or reshaping of the fibers at the points of contact in each case.

**Table 3:** Predicted and measured values for porosity and pore size for tubes fabricated with varied winding angle

Case	Predicted porosity (unbonded) (%)	Predicted porosity (bonded) (%)	Measured porosity (%)	Predicted pore size (×10^−2^ mm^2^)	Measured pore size (×10^−2^ mm^2^)
1a	91.9	83.72	86.72	7.84	6.9 ± 1.9
1b	91.08	82.08	87.23	5.32	5.0 ± 1.1
1c	89.51	78.94	87.32	5.28	4.8 ± 0.6

Also shown in Table 3 are predicted values for pore size which reduce from 7.84 × 10^−2^ mm^2^ to 5.28 × 10^−2^ mm^2^ as the winding angle is increased from 30° to 60°. The average measured values shown in Table 3 demonstrate that pore size reduces (from 6.9 ± 1.9 × 10^−2^ mm^2^ down to 4.8 ± 0.6 × 10^−2^ mm^2^) with increased winding angle. The large variation in the measured values can be attributed to the presence of field disturbances during the electrospinning process as well as repulsive coulombic interactions between charged fibers immediately prior to deposition [47]. However, these results demonstrate that choice of design parameters such as number of fibers, fiber diameter and winding angle can be used to predict tube architecture such as pore size, number and total porosity.

### Mechanical Testing

**Fig. 6:**
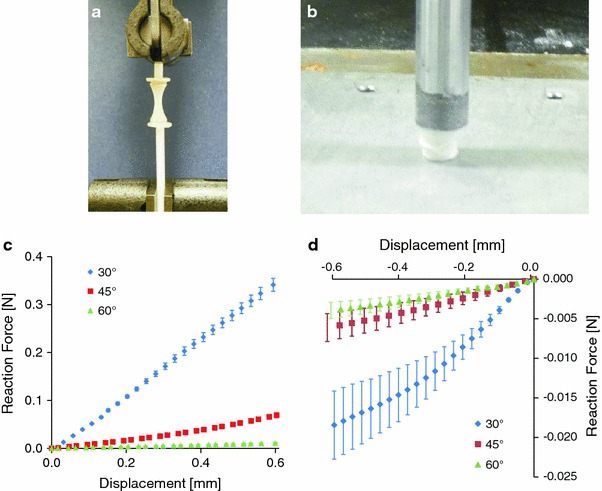
Mechanical testing of melt electrospun wound tubes in **a** tension and **b** in compression (where the onset of buckling is shown). Response of tubes to mechanical loading in **c** tension and **d** compression. Mean values and standard error bars are shown (*n* = 5)

Tensile and compressive testing of melt electrospun tubes is shown in Fig. 6a and b. Figure 6c shows average mechanical response data to uniaxial tensile testing for melt electrospun tubes fabricated with 30°, 45° and 60° winding angles, where at 10% strain the reaction forces are 0.34 ± 0.013 N, 0.07 ± 0.005 N and 0.011 ± 0.002 N, respectively. The reactions are significantly different and stiffness appears linear which suggests that a linear approximation for the material properties of PCL in tension in the FE model is valid over this strain range. Considering the predicted trends in Fig. 3: the uneven difference between the response curves appears closer to the unbonded simulation shown in Fig. 3a where reaction reduces according to a power law as winding angle is increased. That is, the reaction force for 45° is much closer to that for 60° rather than 30° whereas if fibers were totally bonded, the 45° reaction would be expected to lie in between those for the other two winding angles in order to follow the linear reduction in response shown in Fig. 3b. The implication of these results is highlighted by considering the 60° case: the response force is so small it suggests the fibers in the tube are still unentangling and aligning themselves towards the direction of loading at up to 10% strain, rather than reacting as a whole bonded structure. Whereas for a 30° winding angle, the fibers become aligned with the direction of loading sooner and therefore provide a stiffer response. Figure 6d shows responses to uniaxial compression where no significant difference was shown between the reaction force for 45° and 60°. Further, the compressive reaction forces are of an order of magnitude less than for tension as well as nonlinear, which suggests that a mechanism such as buckling is taking place as unbonded fibers unentangle up to 10% strain as observed in Fig. 6b. These results indicate that the choice of a fabrication design parameter such as winding angle significantly affects the mechanical properties of a melt electrospun tube, and that FE simulation is a valid design tool which can be further developed to predict such variations in mechanical behavior as well as the degree of fusion between fibers.

### In Vitro Biocompatibility Assessment

The first step in optimizing TE scaffold design is to understand how scaffold architecture influences cellular activities. From this point of view, solution electrospinning has a number of drawbacks associated with chaotic fiber deposition and thus random assembly characteristic to the process [50]. Therefore, our aim is to translate melt electrospinning, which offers improved control over fiber deposition, into the direct writing of tubular scaffolds based on better control over design parameters. Pore geometry plays a vital role in governing cell infiltration, with the shape of the pore dictating the distribution of cells within, and the resultant pattern of tissue growth. For example, the evaluation of diamond-shaped pores suggests that they are less sensitive to initial conditions of cell attachment than rectangular pores, and thus more effective in guiding engineered tissue cell and collagen orientation [51]. Edwards et al. [23] report that tissue growth can be predicted based on aspects of pore geometry, namely, the angle between crossing fibers and the distance between adjacent fiber crossovers. Tissue lengths at fiber crossovers were found to decrease exponentially with increasing interfiber angle, as well as decrease away from the fiber crossover, with the smallest lengths toward the fiber segment mid-point. Considering this approach in the design of TE scaffolds, it is envisaged that tissue growth may, in part, be controlled by scaffold fiber orientation (dictating the interfiber angle) and packing density (controlling the distance between adjacent fibers). Therefore, the aim of the following in vitro cultures was to demonstrate the biocompatibility of these structures for future exploration into the aforementioned issues.

#### Culture of Human Primary Osteoblasts on Tubular PCL Scaffolds

**Fig. 7:**
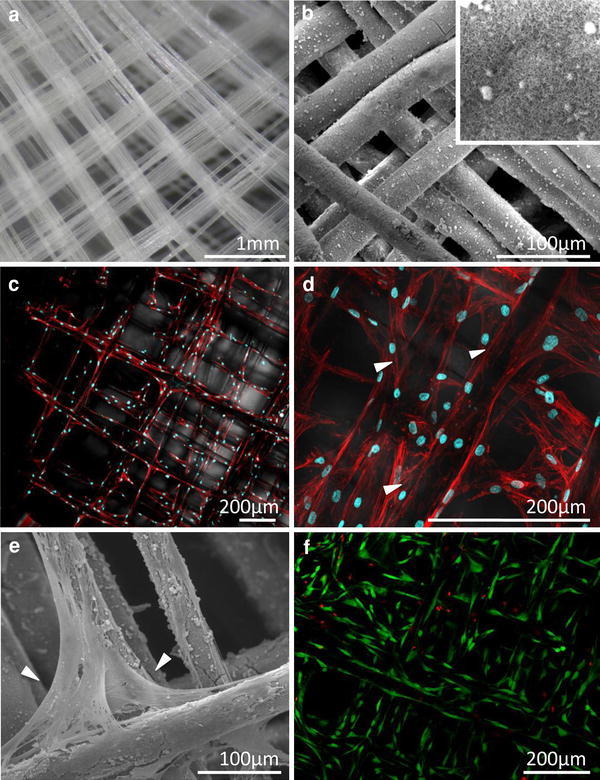
Culture of hOBs on CaP coated electrospun PCL tubular scaffolds. **a** Optical image showing fibers with winding angle of 45° prior to CaP coating. **b** SEM image showing the coated PCL meshes prior to cell seeding. An SEM image taken at higher resolution (*inset* 25 × 25 μm) reveals the microstructure of the biomimetic coating. **c** CLSM 3D projection of hOBs cultured on the scaffold for 2 weeks. Cells were fixed and stained for f-actin (*red*) and nuclei (*blue*). An overlay of the fluorescent and transmission light microscopy image is shown. **d** LSM 3D projection taken at higher magnification [same conditions as in **c**]. **e** SEM image showing hOBs after 2 weeks of culture on the scaffold. *Arrows* in **d** and **e** indicate fiber crossovers where hOBs start to span neighbouring fibers. **f** Live/dead assay by using FDA/PI staining showed that >90% of the hOBs were alive after 2 weeks of culture

To facilitate attachment and growth of hOBs and provide a bone-mimicking crystalline microenvironment, the fibers of melt electrospun PCL tubes (Fig. 7a) were coated with a CaP layer. SEM analysis indicated that an approximately 800 nm thick and relatively uniform coating was deposited by this procedure (Fig. 7b). Higher resolution images (inset in Fig. 7b) revealed that the coated PCL fibers had an increased roughness compared to smooth non-coated fibers (Fig. 4c) which could account for enhanced cell/material interaction. After 14 days of in vitro culture under osteogenic induction, CLSM imaging showed the hOBs were homogeneously distributed over the entire fibrillar network (Fig. 7c). The cells were preferentially orientated along the fibers and exhibited an elongated spreading morphology with extensive actin stress fibrils. Higher resolution CLSM and SEM images show that some hOBs began to span two adjacent fibers at their crossover points, indicated by the arrows in Fig. 7d and e. Generally cells displayed a healthy spreading morphology, which was further confirmed by the high cell viability >90% detected by FDA/PI stainings (Fig. 7f).

#### Culture of Mouse Primary Osteoblasts on Tubular PCL Scaffolds

**Fig. 8:**
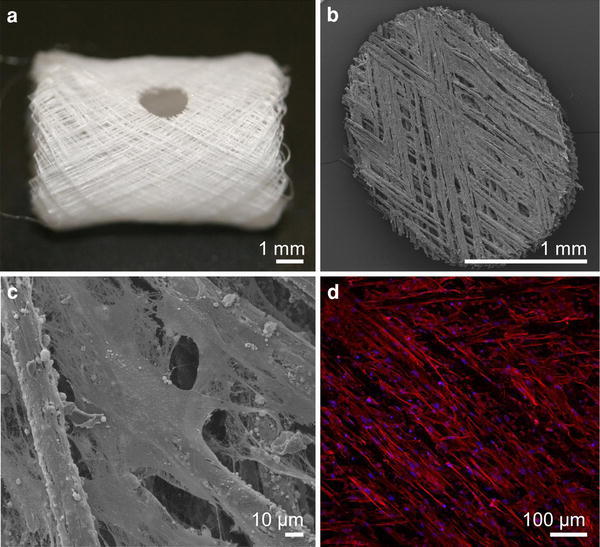
Culture of mOBs on melt electrospun PCL fibers taken from a tube with a winding angle of 30°. mOBs initially showed good attachment and then proliferated over a culture period of 4 weeks. **a** After 4 weeks of culture a 2 mm biopsy punch was used to harvest the specimen shown in **b**–**d**. SEM showed that the mOBs formed a mineralized ECM not only on the scaffold fibers but also inside and across the pore architecture (**b** and **c**). **d** CLSM revealed by using DAPI/Phalloidin staining alignment of mOBs along the fiber axis

One of our future goals is to use the tubular scaffolds to develop an ectopic long bone model in mice to study bone metastases. Similar to hOBs, mOBs showed good attachment and then proliferated over a culture period of 4 weeks. After 4 weeks of culture a 2 mm biopsy punch was used to harvest the specimen (Fig. 8a) shown in Fig. 8. SEM (Fig. 8b, c) showed that the mOBs formed a mineralized extracellular matrix (ECM) not only on the scaffold fibers but also inside and across the pore architecture. DAPI/Phalloidin staining revealed under CLSM alignment of mOBs along the fiber axis of tubular scaffolds (Fig. 8d).

#### Culture of Human Mesothelial Cells on PCL Scaffolds

**Fig. 9:**
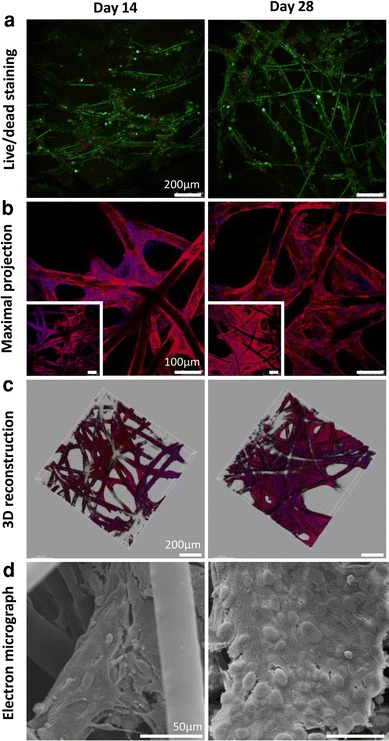
Met-5A cells were seeded on PCL meshes at a density of 2 × 10^5^ cells in 40 μL. Scaffold/cell constructs were imaged every 2 weeks performing cell viability and imaging analyses using CLSM (**a**–**c**) and SEM (**d**). **a** Over the culture period a cell viability of greater than 90% was determined by live/dead staining. **b** Maximal projections of CLSM images (*inset scale bars* 100 μm) revealed an ongoing cell/ECM sheet formation covering more than 75% of the pore space indicated by (**c**) 3D reconstructions. **d** SEM revealed the morphology of Met-5A cells, where sheet formation started on day 14 and continued up to day 28

When Met-5A cells were cultured onto the PCL scaffolds, they were able to proliferate and span pores similarly to the hOBs. Met-5A cells grown up to 28 days on top of PCL meshes were imaged every 2 weeks performing cell viability and imaging analyses using CLSM and SEM. Over the mesh culture period a cell viability of >90% was determined by live/dead staining, exemplifiying the excellent cyto-biocompatility of theses structures (Fig. 9a). Maximal projections of CLSM images (Fig. 9b) revealed an ongoing sheet formation covering more than 75% of the surface as seen in the 3D reconstructions (Fig. 9c). SEM confirmed that the cell sheet formation started 14 days post-seeding and continued up to day 28 (Fig. 9d).

## Summary and Conclusion

Over the last 10 years there have been significant advancements in the field of electrospinning. In spite of this, new breakthroughs in manufacturing are needed to maintain the momentum of the electrospinning field. For the most part, researchers have focused on electrospinning from solution with only a handful attempts to design and fabricate scaffolds from the melt. Our group has made significant progress in the design and fabrication of scaffolds by combining melt electrospinning with a direct writing process, where matching the translation speed of the collector to the speed of the melt electrospinning jet is the key factor which establishes control over the location of fiber deposition in order to write with a continuous line. In this work we were able to accurately deposit melt electrospun fibers and create tubular scaffolds with different fiber winding angles. Control over the relative speed between translation and rotation allowed control over fiber diameter (due to induced drawing) and the winding angle. Combined with the choice of fiber number, these design parameters allowed control over scaffold architecture in terms of number of pores, their size and geometry, as well as total porosity. A computational model was developed and validated against mechanical testing results to show that melt electrospun tubes provide a stiffer response to uniaxial loading when fabricated with a smaller winding angle and no fusion between fibers.

The scaffolds that are used in TE are generally meant to provide provisional substitutes for ECM, providing a temporary structural support combined with specific biochemical signals that encourage cells to create their own ECM environment. However, the ECM is much more than a static mechanical support for tissues. It provides the physical microenvironment of a cell. Cells are not only affected by material composition, but also by the topography and mechanical properties of the scaffold. We have shown in a series of in vitro studies that tubular melt electrospun scaffolds fabricated in a direct writing mode show excellent cell compatibility. Taken together, these in vitro studies demonstrate that hOB, mOB and human mesothelial cells were able to infiltrate entirely the fibrillar scaffolds and that the specific architecture obtained by the combination of melt electrospinning and a direct writing process is favorable for cell spanning between adjacent fibers. Future work of our group is focused on the design and implementation of in vivo studies to further explore the potential of this unique scaffold design concept.

## Electronic Supplementary Material

Below is the link to the electronic supplementary material.

Supplementary material 1 (DOC 1666 kb)
