# Supplementary material for: Design and Fabrication of Tubular Scaffolds via Direct Writing in a Melt Electrospinning Mode
Source: Biointerphases. 2012 Feb 9;7(1-4):1–16. doi: 10.1007/s13758-011-0013-7 (PMC4875147; doi:10.1007/s13758-011-0013-7)

**Supplementary information**

*Tube pore architecture design parameters*

Tube diameter = d_t_

Tube height = h

Fibre diameter = d_f_

Number of fibre pairs = n

Winding angle = β

*Response Parameters*

Circumference = c = πd_t_

Total surface area of a cylinder which tube comprises = a_c_ = ch

Cross sectional area of fibre = A_f_ =
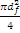


Fibre length = l =
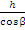


Spacing between fibre centres around circumference = s_cf_ =
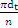


Spacing between fibres around circumference = s_c_ = s_cf_ - f_c_

Where f_c_ = apparent fibre width at circumference =
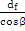


Spacing between parallel fibres = s_f_ = s_cf_cosβ - d_f_

Total number of fibres that fit in on tube = n_f_ =
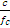
 =
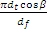


Pore length = l_p_ =
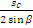


Pore height = h_p_ =
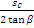


Approximated single pore area = A_p_ =
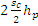
= s_c_h_p_ =
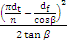


Repeating unit height = h_u_ =
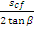


Number of pores / repeating unit = number of fibre pairs = n

Total number of pores = n_p_ =
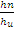


Number of crossover points = n_c_ = n + n_p_

Porosity (bonded fibers):

Total volume of a tube which contains a single layer of fibres = V_tcb_ =
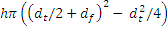


Volume occupied by fibres in this tube = V_f_ =
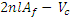


Where V_c_ = the adjusted volume where fibres crossover (without this value the volume where fibres crossover would be included twice rather than once), derived below:

Fibre crossover height = h_fc_ = h_u_ - h_p_

Length of fibre crossover = l_fc_ =
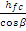


Volume of fibre crossover = V_fc_ = l_fc_A_f_

Therefore, V_c_ = V_fc_(n_p_ + n/2) (the n/2 accounts for the starting row of fibre crossovers will only take up half of the crossover volume)

Porosity (bonded) = ρ_b_ =
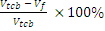


Porosity (unbonded fibers):

Total volume of a tube which contains a single layer of fibres is adjusted so that fiber pairs sit on top of each other rather than fuse = V_tcu_ =
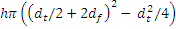


Porosity (unbonded) = ρ_u_ =
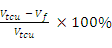

Supplement: Supplementary file 1 — Supplementary material 1 (DOC 1666 kb) [file 13758_2011_13_MOESM1_ESM.doc]
